# How “Neighborhood” Arose, Changed, and Grew: A Bilingual Canadian Story

**DOI:** 10.1177/00961442231170239

**Published:** 2023-05-10

**Authors:** Richard Harris

**Affiliations:** 1 McMaster University, Hamilton, ON, Canada

**Keywords:** neighborhood, quartier, residential area, Canada, Toronto

## Abstract

“Neighborhood” is routinely used when referring to the history of residential areas in North American cities. In fact, it is unclear whether this has always been the preferred term, and how its meaning has changed. A survey of the English- and French-Canadian experience, including a case study of Toronto using digital newspaper files, indicates that in the early twentieth century other terms were common. “Neighborhood” referred primarily to poorer, immigrant districts. Especially since the 1960s, it has been much more commonly used and in a general sense. In that regard, its evolving meaning has converged with the francophone usage of *quartier*. It is only recently that local associations have dropped “ratepayer” from their names in favor of “residents” or, to a lesser extent, “neighborhood.” This now disguises the fact that such associations are dominated by property owners. Getting the language right is important for a clear-eyed understanding of both the past and the present.

Historians, indeed all people of a certain age, know that words can change their meaning. This is obvious when looking back over centuries. No one can speak about Bruges in the 1400s without acknowledging that “city” carried more political weight then than now. But the profile and meaning of words can shift over much shorter periods—decades, or even years. It is easy to lose sight of that fact. Today, for example, we freely speak about “neighborhoods” in early twentieth-century cities as if the past relevance and meaning of the term is obvious. We shouldn’t.

Take New York City. Kenneth Scherzer has argued that most residential areas in Lower Manhattan in the mid-nineteenth century were unrecognized as such, and unlabeled. Working-class residents did not identify, or speak about, neighborhoods, and no one else found it necessary. The term, and its connotations, was introduced rather later, by middle-class epidemiologists and moral reformers. We do not have to go that far back in time to encounter uncertainty. Suleiman Osman has argued that in recent decades Brooklyn gentrifiers have created neighborhood names wholesale, including “Cobble Hill.” Indeed, the entire generic term “Brownstone Brooklyn” was invented in the 1970s. Most intriguingly, Osman argues that before this the borough had “zones” but “no real neighborhoods.” The implication is that to talk about neighborhoods in Lower Manhattan in the 1860s, or in Brooklyn in the 1960s, is anachronistic and potentially misleading.^1^

New York City, of course, has long been typical of nothing. In addition, some might argue that Scherzer and Osman overstate their respective cases. Elsewhere, for example, using Boston’s Jamaica Plain as a case in point, Alexander von Hoffman has argued that, in “the late nineteenth and early twentieth century, neighborhoods served as important centers of gravity within the complex social organization of the city.” In the 1920s, Roderick McKenzie was one of many Chicago sociologists who believed that neighborhoods played a vital role in the local life of the city, albeit one they feared was in decline.^2^ But, if nothing else, the claims of Scherzer and Osman suggest that, instead of making an assumption, we should approach the question with an open mind, looking more closely and systematically at how locals and contemporaries have spoken about residential areas. Surveying the Canadian experience, both anglophone and francophone, and focusing on Toronto as a case study, that is the purpose of this article.

This is not an academic, etymological exercise. If, as I argue, “neighborhood” was once used less often and in different ways than today, we run the risk of misinterpreting the past if we use the term unthinkingly. In the name of tradition, we might also find ourselves sanctifying a word that, for some, has become associated with Not In My Backyard (NIMBY) resistance to intensification. This modern association with a planning goal that is justified in environmental terms would have seemed strange indeed a century ago. In our dialogue with the past, it is always worth using language carefully.

The value of considering Canadian usage in both official languages is that it tests whether long-run trends have crossed a cultural divide. It turns out that they do. Within English Canada, it would be idle to claim that Toronto’s experience has been typical since, with the partial exception of Zane Miller’s observations about Cincinnati, there is no comparable study of any other Canadian or American city. At the very least, however, it is surely interesting. In the 1970s, Toronto overtook Montreal as the largest urban area in Canada and it is currently the sixth largest on the continent, excluding Mexico. Moreover, since the 1980s, it has proudly styled itself as “the city of neighborhoods.” In that respect, it has offered itself as an appropriate test that “neighborhood” has been both a prominent and durable feature of the city. It turns out that neither is the case. A century ago, other terms were more widely used, and “neighborhood” only came into its own in the 1970s. That is also when some residents’ associations began to use the term. The intriguing question is “Why?”

To answer this question, it is helpful to consider the arguments of Christian Topalov. Unlike many prominent French sociologists, not only does Topalov have a grounded historical perspective but he also writes in accessible prose. More to the point, based on his knowledge of the evolution of names employed in various languages to denote the physical features of cities, he has framed a plausible explanation of how names arise.^3^ His framework helps make sense of the evolution of the language deployed in Toronto. It also helps us to speculate about nomenclature in other cities, a task for the conclusion.

## The Prominence of “Neighborhood”

Toronto is not unique, in Canada and beyond, in calling itself the city of neighborhoods. Indeed, that has become something of a fashionable phrase, which is consistent with the observations of Zane Miller. Based in part on his deep understanding of the history of Cincinnati, Miller surveyed the changing role of “neighborhood” in American cities.^4^ He suggested that the concept appeared in the late nineteenth century, an intriguing observation that few others have noted but which is borne out by a British parallel. In the 2000s, Christian Topalov led an international project, *Les Mots de la Ville*, the results being published in a 1489-page volume in 2010. Akin to an encyclopedia, it contains hundreds of entries that track the manner in which urban words have evolved in seven languages. In his entry on “neighbourhood,” the British urban historian David Reeder notes that Charles Booth, who undertook exhaustive surveys of London’s residential areas in the late nineteenth century, used a range of terms, including “district,” “precinct,” and “quarter.”^5^ “Neighbourhood” was largely absent. It is not clear whether he downplayed the word because he preferred more neutral terms—he avoided “slum,” for example—or whether he reckoned that it was not especially common. Miller is more explicit. He judged that, in the United States, “neighborhood” then “flourished” for a couple of decades in the early twentieth century but then “all but disappeared” from the 1920s through the 1940s. This is broadly consistent with usage in Seattle in 1929, as reported in a study that examined the full range of area-based organizations: Although “neighborhood” was quite common, “residents’ “and “citizens’ “associations, as well as “community clubs,” were also well used. It was later, in the early postwar period that, according to Benjamin Looker, as an “ideal for urban living” “neighborhood” “reemerged with a startling intensity.” Miller dates its strong revival in everyday language a little later, to the 1960s, after which it gained greater momentum.^6^

Numbers in Google’s *Ngram Viewer* confirm Reeder’s comments and Miller’s impressions. This software tool, used less frequently by historians than it might be, supports keyword searches of a huge database of works published between 1500 and (currently) 2019.^7^ Usefully, it shows the relative, not absolute, frequency with which words appear, therefore indicating their shifting profile. It is possible to make separate searches on “neighbourhood” and “neighborhood,” and these do yield subtly different results. Although Canadians now supposedly use the British spelling, the American version is the more relevant. It speaks not only to the experience of Americans, but for many decades also of Canadians. American spelling was especially common in the early decades of the twentieth century and persisted occasionally in print journalism into the 1980s. A notable early illustration is *My Neighbor*, published in 1911. Written by J. S. Woodsworth, the founder of the first neighborhood house in Canada and later cofounder of a left-leaning national political party, it made the case that immigrants should be treated, and helped, as equals.^8^ Using “neighbor,” then, an NGram search shows an early peak in usage in the late nineteenth century, a deep interwar lull, and then with rising waves, a peak today.

That pattern is borne out by the writings of Canadian urban experts and historians. The extended interwar trough, for example, is striking. In the midst of the Depression, H. A. Bruce, the lieutenant governor of Ontario, established a Committee on Housing Conditions in Toronto. At a speech he gave in March 1934, which signaled his intention, Bruce contrasted “fine residential areas” with “slum districts,” terms that the report itself echoes, along with “parts of the city.” Apparently, neither Bruce nor members of the committee saw “neighborhood” as a useful generic term. The influential Bruce report described the housing situation in various inner-city “districts” but never used the n-word. The same was true, more surprisingly, of a study of Vancouver completed a decade later by a geographer. Donald Kerr spoke only about “all the residential districts in Vancouver,” while, as late as the mid-1960s, Barry Mayhew’s survey for Vancouver’s United Community Services spoke of “local areas.” None mentioned neighborhoods.^9^

Canadian and other historians, are usually sensitive to past usage and have followed that cue. Of course, we all see the past through the eyes of the present and urban historical researchers may unconsciously use terms that contemporaries would not. One way of minimizing this problem is to consider the writings of those who did not think of themselves as “urban,” and who therefore would not have imbibed the modern social science discourse about cities. The results are sobering to those who believe that spatial patterns matter. For example, two histories of Vancouver written by nonurban historians make only casual reference to residential areas, and none to “neighbourhoods.”^10^ But of course, some non-urbanists are more sensitive to the social geography of cities than others. One is Terry Copp, a social historian and author of a respected study of poverty in Montreal before the Depression. At the time, no Canadian city was larger, or contained more striking socio-geographical contrasts. Copp acknowledged this. He took his cue from Herbert Ames, the late nineteenth-century author of a detailed study of several of the poorer, working-class districts of the city that had been inspired by the work of Booth in Britain. Early on, Copp refers in turn to “district,” “quarters,” “*quartiers*,” “section,” “ward,” “locality,” and “area” before first mentioning “neighbourhood.” He then reverts to “section,” “wards,” “area,” and “district,” adding “parts of the city,” all on page 25. His varied usage implies uncertainty about what language to use. Writing at a time when a neighborhood movement was shaping urban politics in many Canadian cities, Copp was respecting contemporary usage, or at least its anglophone version. In the depths of the Depression, when the Montreal Board of Trade’s Civic Improvement League (1935) reported on housing conditions, it, too, ignored “neighbourhood.”^11^

The language of writers such as Copp and Kerr and of the contemporary experts who wrote reports in Toronto and Montreal are surely indicative. But what matters most in the long run is the language of the street.^12^ We need to pay attention above all to popular usage.

## Popular Usage in English: The Case of Toronto

The best way of capturing the way people in the past used to speak about the areas in which they lived is to read the daily newspapers. These reported local goings-on and in everyday language, or else they would soon be out of business. Fortunately, the searchable full-text of two Canadian English-language dailies are available since the turn of the twentieth century. The *Toronto Star* has been the most widely read locally. Unfortunately, its historical database does not reach back into the nineteenth century. More importantly, its search tool is limited.^13^ For that reason, only very selective use of this source has been made here. Instead, this analysis relies on the Toronto *Globe*, which merged with the *Mail* in 1936 to form the *Globe and Mail*, currently available on ProQuest from 1844 to 2019.^14^ True, the *Globe and Mail*’s readership has always been biased toward the middle and upper classes. Jean Bradley’s memoir underlines the point. In suburban working-class Newtonbrook between the wars, she recalls that “we cut the *Daily Star*, the *Telegram*, or the local weekly *Enterprise*, into small squares to hang on a nail in the outhouse” [but] “I never saw *The Globe and Mail* used this way . . . presumably its subscribers had indoor plumbing—although,” she added mischievously, “they may have used it at summer cottages in the Muskoka Lakes.”^15^ Point taken. But, uniquely, the historical depth of its database, coupled with a versatile search engine, makes it possible to explore trends in urban usage in Toronto with great subtlety and more so than for any other Canadian city. In the post–World War II (WWII) era, it broadened its geographical scope, eventually developing regional editions, and it is today the leading national daily. But it has always had a local bias. Particularly, in earlier decades, references to residential areas in its main, Ontario, edition pertain predominantly to Toronto, which in the 1970s overtook Montreal to become Canada’s largest metropolitan area.

### How the Database Was Used

For present purposes, a variety of keyword searches were undertaken of articles published in the *Globe (and Mail)* from 1880 to 2019. Initially, a variety of words and phrases were explored, but confined to only those that might plausibly pertain to a range of residential areas.^16^ For that reason, words such as “slum” or “suburb” that have always denoted a specific type of area were not included.^17^ It emerged that, in addition to “neighbourhood,” two terms have been widely used at various times since the 1880s: “residential district” and “residential area.”

Tracking the prominence, and relative importance, of these three terms was not straightforward. An obvious problem was that “neighbourhood” was used in a variety of contexts, many having nothing to do with residential areas. For example, “in the neighborhood of” has both literal and metaphorical senses. Full-text searches yielded a large majority of irrelevant or misleading references. By confining searches to the titles of articles, it was possible to eliminate almost all such material. Conversely, references to “residential area” and residential district’ were invariably relevant but rarely appeared in titles. Here, full-text searches were used. This means that the raw counts for these two terms are not directly comparable with those for “neighbourhood.” However, indexed values that show changes in their relative profile reveal notable trends.

The calculation of index values itself required some care. Unlike the *Ngram Viewer*, ProQuest’s search tool does not adjust for the relative frequency with which words appear. Simple counts would be misleading because over the decades the newspaper varied in length. One way to assess this is to use a consistently common word as a control. “And” was used. It appeared 36,581 times in the *Globe* in the 1860s, rising steadily through 126,166 in the 1890s before leveling off in the 1920s, when the count was 423,637 (Table 1). After minor fluctuations, it peaked at 493,930 in 1980s before falling in the 2010s as the daily newspaper slimmed down. Although the search tool suggests that the database covers the entire decade of the 2010s, at the time the research was undertaken it only covered up to and including 2017. Accordingly, counts for the 2010s have been multiplied by 10/8 to render them comparable to previous decades. Indexed, using the 1950s as a base, values ranged from a high of 123 in the 1980s to lows of 24 in the 1880s and 72 in the 2010s.^18^

**Table 1.** The Indexing of “Neighborhood.” Raw counts for “and” are based on the full text of articles. Those of “neighbourhood” are based on titles only. The search engine automatically includes British and American spelling of “‘neighbourhood.”.

	Raw counts		Counts indexed		Adjusted index for “neighbourhood”
	“And”	“Neighbourhood”	“And”	“Neighbourhood”	
1880s	96,085	28	24	108	450
1890s	126,166	37	32	142	444
1900s	200,262	28	50	108	216
1910s	289,188	80	72	308	427
1920s	423,637	128	105	492	469
1930s	440,802	68	110	261	237
1940s	456,626	31	114	119	105
1950s	400,491	26	100	100	100
1960s	441,859	24	110	92	84
1970s	481,997	73	120	281	234
1980s	443,930	74	123	285	232
1990s	387,973	54	97	592	610
2000s	398,053	379	99	1,459	1,474
2010s	231,519	311	—	—	—
2010s adj^a^	289,399	389	72	1,495	2,076

Source: ProQuest *Globe and Mail* database.

a Counts adjusted to obtain comparable estimate for whole decade.

Using the counts of “and,” the results for other keywords were standardized. This made it possible to provide a meaningful interpretation of historical trends. Two approaches were taken. The first was to construct an adjusted index for each keyword. Again, using the 1950s as a base, interim index values were calculated, and then adjusted by the “and” index. The procedure for “neighbourhood” illustrates the method (Table 1). In the 1950s, 26 articles in the *Globe and Mail* contained “neighbourhood” in the title, receiving an interim index value of 1.00. There were 37 such articles in the 1890s, earning an interim value of 1.42, and 379 in the 2000s, earning an interim value of 14.58. But a comparison of these three values—in historical sequence 1.42, 1.00, and 14.59—would be misleading because, as noted above, the newspaper was much shorter in the 1890s than in later decades: index values for “and” in those decades were 0.32, 1.00, and 0.99. And so, to enable meaningful comparison, the interim values for neighborhood were adjusted by those for “and.” For the three decades in question, this yielded a sequence of 4.44, 1.00, and 14.74. This procedure made no significant difference to a comparison of the 1950s with the 2000s but made a substantial difference to our understanding of all decades prior to the 1920s, and also the 2010s. The same procedure was adopted for “residential district” and “residential area.”

A second approach retained the raw counts, suitably adjusted. Index values give a clear impression of historical trends but disguise the absolute numbers on which they are based. Those numbers themselves are also of interest. For that reason, in the second approach, absolute numbers (respectively, 37, 26, and 379) were adjusted by dividing by the appropriate index values for “and,” in this case 0.32, 1.00, and 0.99. The results are counts, in this case 115, 26, and 382, which might be termed hypothetical (Table 2). Their purpose is to indicate that, if the newspaper in the 1890s had been as substantial as it was in the 1950s then there would have been 115 articles with “neighbourhood” in the title, not 37. Obviously, such numbers need to be interpreted with care, especially before the 1920s, after which the discrepancy between reported and adjusted counts is generally quite small.

**Table 2.** The Language of Neighborhood in the *Globe and Mail*, 1880 to 2019 Counts.

	Raw counts			Adjusted counts^a^		
	RD	RA	N	RD	RA	N
1880s	2	—	28	8	—	108
1890s	13	—	37	41	—	115
1900s	78	4	28	156	8	56
1910s	72	10	80	100	14	111
1920s	93	34	128	89	32	122
1930s	64	40	68	58	36	62
1940s	67	62	31	59	54	27
1950s	92	246	26	92	246	26
1960s	66	331	24	60	301	22
1970s	42	271	73	35	226	61
1980s	55	267	74	45	217	60
1990s	21	158	154	22	163	159
2000s	22	165	379	22	166	382
2010s	16	65	311	22	90	432
2010s adj^b^	20	81	389	28	112	540

Source: ProQuest *Globe and Mail* database.

Note: RD = residential district (full text search); RA = residential area (full text search); N = neighbourhood (title search only).

a Counts adjusted by the relative frequency of “and” in full text.

b Counts adjusted to obtain comparable estimate for whole decade.

### What the Newspaper Evidence Shows

Suitably adjusted, the newspaper evidence shows some notable trends and shifts. “Residential district” peaked early, in the 1900s, fluctuated, and then went into decline after the 1950s (Table 2). “Residential area” gathered momentum over the first half of the twentieth century, peaking in the 1960s before falling thereafter. In contrast, confirming the impression of Zane Miller and the evidence of NGram, the pattern for “neighbourhood” has been bimodal, peaking in the late nineteenth and early twentieth centuries, falling through the 1940s, 1950s, and 1960s, before coming back strongly since the 1970s. Indeed, the switch can hardly be overstated. In recent decades, “neighborhood” has been referenced more frequently in the *titles* of articles than the other terms appear in the full text. Because title searches are not supported by ProQuest’s *Star* search tool, precise comparison is impossible, but it is notable that references to “neighbourhood” in its full text moved in parallel with the *Globe*’s titles. After a low peak in the 1920s and 1930s, they began rising again in the 1970s, taking a steady eightfold jump by the 2010s. “Neighbourhood” is now overwhelmingly dominant.

This points to a curious, and continuing, disjuncture between expert and popular interest. In the 1920s, among urban sociologists, in particular, there was a surge of academic interest in neighborhoods in the United States, which found some echo in Canada at a time when popular usage in both countries was on the decline. At the time, there was no academic journal for sociologists (or urbanists) in Canada, but the *American Journal of Sociology* (1894–) shows a surge of interest in neighborhoods through the 1920s and 1930s, coinciding with the interwar lull documented in NGram. The correspondence between popular and academic interest has not been much stronger in the postwar period. In Canada, in the early postwar decades, three academic journals were founded that contained a significant proportion of the academic discussion of neighborhoods: the *Canadian Geographer* (*CG*; 1954–), the *Canadian Review of Sociology and Anthropology* (1964–), and the *Canadian Journal of Sociology* (*CJS;* 1975–). Online keyword searches of the titles and abstracts of articles published in these journals are possible. The trend in the *CG*—a tenfold increase since the 1950s—tracks popular usage, as reported in Toronto’s two dailies, but the two sociological journals show a gentle increase to the 1990s, followed by a marked decline. The *American Journal of Sociology* showed likewise. It is beyond the scope of this article to explore why trends in academic discussion have departed from wider trends. It may be that many researchers have bought in to the idea, deplored by Robert Sampson, that globalization in the digital era has eroded the significance of place.^19^ But, if nothing else, the disjuncture between expert and lay conversations underlines the importance of considering both.

What does this mean? Counts alone raise more questions than they provide answers: they say nothing about the meaning of words and phrases, and how those meanings changed. For that, it was necessary to read the articles in question, or at least a representative sample. I adopted an informal procedure. In each case, decades were selected that were especially notable for each word or phrase—when they appeared, were unusually common, or went into decline—and then I read those that ProQuest flagged as most “relevant” until a clear pattern of meaning or association emerged. The alternative, of reading all articles, was infeasible: life is too short.

This selective reading of the *Globe*’s articles shows that the meaning of these three terms changed, and sometimes overlapped, to only a limited degree, while that of “neighbourhood” has actually shifted. The origin and evolution in usage of each term illustrates the usefulness of Christian Topalov’s argument about the naming process. Typically, he argues, a word will emerge, or be reconceptualized, when three conditions are met: a new social circumstance has arisen for which there is no existing term, there is a linguistic resource available to fill it, and there are one or more interest groups who wish to promote it.^20^ “Residential district” fits the bill. Responding to public pressure following a major fire, in 1904 a new bylaw consolidated and expanded the scope of land-use regulations. It enabled residents to petition the City to exclude nonresidential uses from defined areas—de facto zoning.^21^ Residents organized, with effect, at scales ranging from the block to fair-sized districts. “Residential district” denoted the areas affected. Here then, combining common terms, was a phrase that responded to new conditions and which was seized upon by an interest group or, strictly, a growing number of interest groups, each defined by place of residence.

Within twenty years, residential districts covered much of the city. Lobbying efforts were often reported. In June 1905, for example, a deputation to the recently established Board of Control asked that the area between Yonge and Spadina, College to Bloor, should be designated, as a newspaper headline declared, “a residential district.” Residents had been goaded by a proposed stable. Such lobbying, and usage, persisted until in 1954 a comprehensive zoning bylaw rendered it moot. By then, it had acquired social connotations. In August of 1951, for example, observers were wringing their hands at the deterioration of the area that included Jarvis street, once “Toronto’s finest residential district.”^22^ Something more than mere land use was implied here.

Some residents wanted more than this, and refined the terminology, and meaning. In response to the city’s first apartment boom, and to local pressure, the municipality prohibited such structures except on major streets. In 1921, it allowed the identification of areas for “private dwellings” only, these in turn being subdivided into “detached” and “other.”^23^ “Area,” then, as opposed to “district,” came to imply greater exclusivity and, again, with social connotations. In 1925, for example, a journalist waxed eloquent about North Toronto as an “exclusive residential area to particular people.” But, perhaps because the language was so similar, the terms were occasionally used interchangeably. For example, when the area’s annexation to the City was impending, an article entitled “North Toronto is a residential area” reported that the Toronto Civic Improvement Committee was asking to have it designated as “a residential district.”^24^ Here, the more exclusive form of land use restriction was clearly implied.

By the mid-1950s, however, “area” was displacing “district” to denote any area that was zoned residential in any way. The case of a frustrated music teacher illustrated this in 1954. On her behalf, and that of its other 1199 members in Toronto, the Ontario Registered Music Teachers Association argued that since anyone could play their piano at home, this instructor should be allowed to move her business from a commercial into a “residential area.” Such usage, along with its social connotations, has occasionally persisted to this day. In 2017, for example, it was reported that Maha’s Fine Egyptian Cuisine on Greenwood Avenue was “tucked in a residential area.”^25^ Since the 1960s, however, this term has been overtaken, and then lapped several times, by “neighbourhood.”

Although “neighbourhood” implies a mainly residential area, usage in the *Globe (and Mail)* underlines that it has always embraced other land uses, notably schools, religious institutions, and commercial operations such as restaurants . . . or music studios. The wrinkle is that its prominence and connotations have changed. In the late 1800s, the term was rarely used and, even then, the word often implied “in the vicinity of.” For example, an article using “neighbourhood” in the title referred to the region around Kingston, a small city more than a hundred miles from Toronto. This is consistent with way Charles Booth gave the term no special attention in his surveys of London. In the early 1900s, however, the word began to be used more exclusively for residential areas, as when reference was made to a new Methodist church in the “Don neighborhood.”^26^ But it was mainly associated with poorer districts. From the 1890s to the 1940s, between 13 percent and 16 percent of “neighbourhood” articles also contained references to immigrants or the poor (Table 3).

**Table 3.** The Associations of “Neighborhood.” The proportion of articles with “neighbourhood” in the title that contain the following words in the full text.

	No. of articles	“Planner” or “planning”	“Worker”	“Poor” or “immigrant”
1880s	28	0	0	25
1890s	37	0	8	16
1900s	28	0	4	14
1910s	80	3	33	13
1920s	128	2	56	13
1930s	68	7	74	15
1940s	31	10	36	13
1950s	26	12	15	8
1960s	24	25	8	8
1970s	73	38	11	12
1980s	74	23	5	12
1990s	154	12	14	16
2000s	379	14	15	11
2010s	311	21	10	10
2010s adj^a^	389	—	—	—

Source: ProQuest *Globe and Mail* database.

a Counts adjusted to obtain comparable estimate for whole decade.

An even stronger association was with those who helped people in need. In the early twentieth century, the most active organizations promoting this were the Christian churches that carried forward the social gospel movement.^27^ The most prominent leader was J. S. Woodsworth, a Methodist minister who established a settlement house in Winnipeg, Manitoba. Inspired by this example, several settlements were soon established in Toronto’s low-income areas, and these became bases from which volunteer “neighbourhood workers” provided services and supports. In the 1910s, references to these workers occurred in 33 percent of all articles with “neighbourhood” in the title, a proportion that rose to 56 percent in the 1920s, when the number of such articles first peaked. The association with immigrants was strong. A typical early article noted that volunteers were “making Canadians” out of immigrant children. In time, the emphasis broadened somewhat, following the creation of a citywide Neighbourhood Workers’ Association (NWA) in 1914. By the mid-1920s, the NWA’s annual presentation claimed that “all over the city a tremendous work is being carried on by volunteer assistants.”^28^ This might suggest that “neighbourhood” was coming into general usage. Not so. The NWA received reports from several districts, but entire sectors of the city, including North Toronto, were absent. The NWA’s focus was on needy households in “slum areas,” whether or not occupied by “newcomers” (ibid.). The connection between “neighbourhood” and volunteer workers tightened still further during the Depression. It is surely no coincidence that the 1920s and 1930s were also the period when, judging from full-text references, the association of “neighbour” and “church” was unusually strong. This association persisted into the 1950s but soon followed “neighbourhood worker” in a steep downward trajectory (Table 3).^29^ For the first half of the twentieth century, in Toronto at any rate, “neighbourhood” was associated with poverty. It had been seized on, initially by Christians, reformers, and the press, but then more generally to denote a particular type of urban settlement.

Since 1945, circumstances, and with them social pressures, changed, and so this term came to denote all types of residential areas, including a range of land uses. Indeed, commercial activity serving local needs came to be seen as intrinsic, essential even. In 1983, an article by Judith Knelman spelt this out. She praised Riverdale, with its Danforth Avenue, as “a neighbourhood of bustling markets and residential streets,” along with parks and convenient transit service.^30^ Such an article could not have appeared a generation earlier, and would have been inconceivable before WWII. “Neighbourhood” had come to embrace something more inclusive than “residential area” or “district,” and it overwhelmed those terms. Today, its warm, inclusive meaning is ubiquitous, but it was not always that way.

If “neighbourhood” overtook other terms, it also absorbed the connotation of defending residential space. The growth of municipal planning after 1945 saw the creation of planning districts and a comprehensive zoning plan. “Neighbourhood” was appropriated by planners and became associated with the mechanisms of land use regulation. The proportion of “neighbourhood” articles with “planner” or “planning” in the text rose slowly from the 1930s and then jumped in the 1960s (Table 3). This reflected a wave of grassroots activism. Coupled with the City Planning Board’s decision to seek citizen input by holding neighborhood meetings, this nudged the proportion even higher in the 1970s.^31^ This surge settled back, but has lately revived as inner districts have come under pressure for redevelopment. There has always been a tension between accommodating local preferences and doing what is deemed best for the city as a whole: in that respect, land use planning is a political balancing act. For that reason, as columnist Alex Bozikovic notes about neighborhood resistance to densification in the Annex, a middle-class inner-city area, “the planning system is maddeningly ambiguous.”^32^

In a general way, today, the defense of residential space against other “incompatible” uses is similar to the opposition to apartments that arose in the early 1900s. But more people now care, and they care more because the stakes are higher. In the early 1900s, about a third of households owned their own home; the average neighborhood was one of tenants.^33^ Today, the rate fluctuates around two thirds, albeit lower in some inner districts, so that many neighborhoods are dominated—numerically, socially, and politically—by homeowners. Just as importantly, the stakes associated with owning a home have risen, above all financially. The significance of these shifts for neighborhoods is clear. In the early decades of the twentieth century, hardly any articles with neighborhood in the title made mention of homeowners. By the 2010s, a third did. References to “real estate” have followed a similar trend.^34^ It is no coincidence that “NIMBY” first appeared in the *Globe and Mail* in 1982, and then took off. “Neighborhood” has inherited the wrangles over land use that were once encompassed by “residential district” and “residential area.” It is because these matter more now than ever before that references to neighborhood have reached new heights.

## Popular Usage in Francophone Quebec

As Terry Copp’s writing suggests, anglophone usage in Montreal has generally been consistent with that in English Canada. But what of francophones in Québec, a province that has famously contained two cultural solitudes? The meanings of related terms in French have been rooted in a different cultural tradition and sustained by distinctive institutions, notably the Catholic church. However, although the starting points were different, lately there has been a notable convergence.

Historically, the most important neighborhood-scale entities were Catholic parishes. Colonial administrators had delegated to them many civil and territorial responsibilities.^35^ The church loomed large, literally: Mark Twain supposedly claimed that it was impossible to throw a stone in Montreal without breaking a church window. Straddling the nineteenth and twentieth centuries, Lucia Ferretti’s study of a poor Montreal parish underlines the pervasive role that it played in local life. Apart from spiritual succor, it provided community along with social services. Often associated with the *paroisses* were local *caisses populaires* (credit unions). Almost as sharply defined as modern local planning areas, *paroisses* once provided an all-encompassing frame to local life. Ferretti has suggested that, although historically the Catholic church had deep rural roots, it also undertook a “creative and functional response” to new urban conditions, putting itself “au coeur des relations sociales.” The church’s role waned slowly and steadily after 1914 as “organizations, receipts, participation in lay groups, everything, now, went into decline.”^36^ But it remained important into the early post-WWII years. In Quebec’s Lower Town, for example, one parish maintained and took on a range of pastoral activities. For example, eight *vicaires* were each made responsible for a subarea, where they made calls to the sick. Indeed, Nancy Christie and Michael Gauvreau have argued that, for a while in Quebec, the church was again “highly effective” in responding to rural-urban migration, creating 491 new parishes, 1940 to 1968, and “reinvigorating the idea of the parish as an urban community centre.”^37^

Complementing the parish, at the smallest scale, has been the language and practice of *voisinage*, which speaks to the immediate environs of the home and of social connections with immediate neighbours, *voisins*.^38^ The concept of having “good neighbours” exists in English-Canada, being implied in the Neighbourhood Watch groups that since 1968 have kept eye on the street. However, there is no exact English-language equivalent to “voisinage.” This is unfortunate because personal accounts and interviews indicate that urban residents care most about their immediate neighbors and more about the block they live on than about any larger area.^39^ This was most obviously true in the past, when neighborhoods were walkable. Ray Godrey, who grew up on Harbord Street in downtown Toronto in the 1940s, recalled that “there was a doctor’s house on the corner, a general store on another corner two blocks away, a bakery, a Chinese laundry, a shoe repair shop,” in sum “a kind of neighbourhood one seldom sees in Toronto any more.”^40^ Even today, as NIMBY implies, we are very alert to those who live close by. However, except in cities such as Chicago that have block clubs, there is no easy way to quantify the significance of this more intimate scale of experience.^41^ Instead, “neighbourhood” seems always to have denoted a larger, if ill-defined area, albeit one that rarely reaches beyond about eight blocks in any direction.

The closest in Canadian French to the current meaning of “neighbourhood” has long been *quartier*, but the trajectory has differed. The word comes above all from Paris; other French cities used varied terms. There, and then in Quebec, *quartier* has always been significant but at a larger scale, as shown by Dale Gilbert’s study of Saint-Sauveur in Québec City in the middle decades of the twentieth century. In the 1800s, *quartiers* were equivalent to wards: their residents elected politicians. Although they lost that power, they remained as administrative units and eventually became *quartiers de planification*. When in 1973 the federal government introduced a Neighbourhood Improvement Program, in Québec it was known as the *Programme d’amélioration de quartier*. However, the term had social significance, too. In *Bonheur d’occasion*, an early, classic Canadian novel set in Montreal’s St. Henri ward, Gabrielle Roy uses the term 120 times, gesturing toward an administrative division but emphasizing its social meaning.^42^

Increasingly, from the 1960s, and indeed in parallel to the experience in France, the social aspects of the term have become more important.^43^ This is acknowledged in planning documents. In 1992, the City’s first Master Plan subdivided all boroughs into planning districts, emphasizing that all “are living environments [‘*milieu de vie*’] with very distinct character,” that are “not limited to the quality of dwellings.” The Plan recognized the role of parks, local services, and, in the same spirit as Toronto’s Judith Knelman, the dynamism of commercial streets. Indeed, later documents treat *quartier* and *milieu de vie* as synonymous. The revised plan insisted that these *milieux de ville* were “diversified and complete living environments.” A transportation document suggested that “for a lot of Montréalers, the term *quartier* refers to the traditional notions of *paroisse* and *voisinage*, that we also call *milieu de vie* in reference to the lived space of the majority of the members of the community.”^44^ The conflation of *quartier* with *paroisse* and *voisinage* is puzzling, but the spirit was summarized by Annick Germain: *quartier* “is not an institutional space but a social one.”^45^

That is surely going too far; it is both. And so, there has been a convergence of meaning across linguistic solitudes. For France, Christine Lamarre has acknowledged that, in its social aspects “*quartier* se rapproche de *voisinage* ou du *neighbourhood* anglais” but in Canada, the coming together of French and English terminology seems to have been overlooked.^46^ Both *quartier* and “neighbourhood” have become portmanteau terms, juggling rather than smoothly integrating the planning and social functions. To many, their gesture toward inclusivity is part of their appeal but so, in practice, is the fact that they primarily represent the interests of property owners. In recent decades, both have grown into unprecedented prominence, above all because they speak for stakes that are grounded in real estate.

## How Local Associations Named Themselves

So, if those are the meanings associated with the various terms that have been used to refer to local areas in the city, how have their residents presented themselves to the outside world, including other urban residents and their local municipalities? Here there is an irony, verging on a paradox. In early decades, when home ownership was less common and mattered less than it does now, residents were candid about who their associations spoke for. Today, when the pressures and financial stakes have never been higher, ambiguity reigns.

Until the 1960s, overwhelmingly, local organizations described themselves as ratepayers, by which they meant payers of property taxes (Table 4). In one sense, this was always a misleading term because tenants pay those taxes too, indirectly through their landlords. However, for many decades, tenants were not allowed to vote in civic elections and “ratepayer” had a certain honesty: Property owners, and especially homeowners, have always been the mainstay of local associations. This was certainly true in the early decades of the twentieth century. As Mary Clarke observed in 1917, when speaking about a downtown immigrant district occupied largely by tenants, ratepayer groups “do not exist in the district where the need for them is greatest.”^47^

**Table 4.** The Language of Residential Organizations in the *Globe and Mail*, 1880 to 2019: Counts.

	Raw counts			Adjusted counts^a^		
	RA	ResA	NA	RA	ResA	NA
1880s	0	0	0	0	0	0
1890s	151	3	0	472	9	0
1900s	66	1	0	132	2	0
1910s	268	1	0	372	1	0
1920s	667	0	0	635	0	0
1930s	402	1	0	365	1	0
1940s	102	7	1	90	6	1
1950s	360	19	0	360	19	0
1960s	339	110	0	308	100	0
1970s	221	206	0	184	172	0
1980s	117	230	1	95	187	1
1990s	24	54	4	25	56	4
2000s	31	70	15	31	71	15
2010s	9	46	18	16	79	31
2010s adj^b^	11	58	23	20	99	39

Source: ProQuest *Globe and Mail* database.

Note: RA = ratepayer associations; ResA = residents’ associations; NA = neighbourhood associations.

a Counts adjusted by the relative frequency of “and” in full text.

b Counts adjusted to obtain comparable estimate for whole decade.

In Toronto, such groups were organized at the ward scale in the late nineteenth century and were part of a citywide organization. They became more local from 1904, after a Board of Control was established, in part to hear the petitions of local residents who wished to lobby for services or for restrictions on land use. In this manner, until the 1950s, ratepayer associations, such as the one established in the Annex in 1923, defended the interests of property owners in the more affluent parts of the city. Similar organizations have been documented and, in much the same period, in many other Canadian cities, including Hamilton, Vancouver, and Berlin, soon to be renamed Kitchener, after a British army officer and colonial administrator.^48^

Inevitably, ratepayer group activities flagged during WWII, but regained momentum in the early postwar decades. The Annex Ratepayers, for example, was greatly revived; others, such as the Durand Ratepayers’ Association in Hamilton, were established.^49^ By the late 1960s, however, tenants had the municipal vote. “Ratepayer” began to acquire exclusionary overtones that a growing number of residents, including newly militant tenants, found objectionable. More groups chose to signal their inclusivity and, by the 1980s, “residents ” associations had become the preferred term (Table 4). In that decade, a small, but steadily growing, number chose “neighborhood” instead of “residents.” It is surprising that there were not more. In the late 1960s and 1970s, in Toronto as across Canada and the United States, there was a wave of neighborhood-based activity. By the late 1970s, as Richard White, the historian of postwar planning in Toronto, stated, the city’s “guiding principle was that neighborhoods should decide for themselves what was best.” Within a few years, Stephen McLaughlin, the City’s Commissioner of Planning and Development was claiming that “neighbourhoods”—not the hubs that made Toronto the financial and cultural capitals of English Canada—“are Toronto’s great strengths.” Regardless, whether styling themselves as residents or as neighbors, Torontonians were implying a degree of local-scale inclusivity that almost never existed in practice. Behind the label, it was a cohort of increasingly anxious property owners who were shaping the politics of land use.^50^

Versions of the same changes were happening in other Canadian cities, although later in Montreal, in part because a majority of its residents were tenants. There, grassroots opposition to centralized civic decision-making developed in the 1970s but it was not until the end of the decade that a “localist” turn began to give a meaningful role to local residents and their organizations.^51^ Eventually, when a new slate of councilors was elected in 1986, some powers were decentralized to *quartiers*. By the late 1990s and early 2000s, a new cultural plan declared that “citizen participation in cultural life starts in the neighbourhood,” while the goal of transportation was defined as “restoring the appropriate quality of life to Montreal’s residential neighbourhoods.”^52^ Here *quartier*, similar to “resident” and “neighborhood,” sounded good, but glossed over the interests that had always shaped the residential parts of the city.

## Concluding Discussion

In Toronto, over the past century or so, the meaning of “neighbourhood” has changed, helping to make it a much more important term. When referring to the residential parts of the city, a century ago it denoted the poorest districts, especially those occupied by immigrants. Other terms, notably “residential district” and “residential area,” were employed much more widely although, because they denoted areas subject to land use restrictions, even they were not universal. No one, anywhere, spoke of “neighborhood associations.” Things began to change after 1945, and with gathering momentum from the 1970s. “Neighborhood” acquired connotations of grassroots democracy and local community, largely displacing other ways of referring to the residential parts of the city and becoming part of the city’s self-image. Lately, with house price inflation and vigorous discussions of the need for densification, there has been growing recognition of the downsides of this decades-long trend. But “neighbourhood” still rules and, with it, the interests of those who once happily described themselves as ratepayers.

Some important details in Toronto’s story are unique. The bylaw changes that were made in 1904, partly in response to a major downtown fire, shaped evolving land use and political possibilities that ratepayers and residents then seized. Their activities, and the terminology they adopted, bequeathed a particular discourse to later generations. However, eventually, “neighbourhood” asserted itself. It may be that Toronto was untypical of Canadian cities, and that in this regard, as in various others, Canadian and U.S. cities differed. But Toronto’s trajectory has close parallels with that described by Zane Miller, and raises a question that others should consider.

This account should obviously not be taken to imply that “neighborhood” had limited significance in the English language before the 1880s, still less that it had always been associated with poverty. The Oxford English Dictionary makes it clear that the term had been in use for centuries. Indeed, long before Chicago sociologists discussed the issue, in 1590, Thomas Nashe fretted that neighborhoods were effectively “dead and buried.”^53^ He was not thinking about slums. The message is simple: The profile and connotations of “neighborhood” have been changing for a very long time.

This matters. To be sure, a century ago, even in 1590, residential areas had many of the characteristics that we associate with neighborhoods today. But they were also different, and talked about differently, too. If, unthinkingly, we speak about them in our everyday language, we may impose inappropriate connotations while missing others that matter. Today, neighborhoods have market value and social meanings that are specific to our time. By implying that those have always existed, we may inadvertently strengthen the claims of self-styled defenders of neighborhoods, whose arguments serve to maintain a status quo that no longer serves our collective needs. We need to be clear-eyed about the present, and also the past.

## Notes

1. Kenneth A. Scherzer, *The Unbounded Community. Neighborhood Life and Social Structure in New York City, 1830-1875* (Durham, NC: Duke University Press, 1992); Suleiman Osman, *The Invention of Brownstone Brooklyn. Gentrification and the Search for Authenticity in Postwar New York* (New York: Oxford University Press, 2011), 19, 22.

2. Alexander von Hoffman, *Local Attachments. The Making of an American Urban Neighborhood* (Baltimore, MD: Johns Hopkins University Press, 1994), xxii; Roderick McKenzie, “The Neighborhood. A Study of Local Life in the City of Columbus, Ohio,” *American Journal of Sociology* 27, no. 2 (1921): 145-168; 27, no. 3 (1921): 344-363.

3. Christian Topalov, “The Naming Process,” in *What’s in a Name? Talking About Urban Peripheries*, ed. Richard Harris and Charlotte Vorms (Toronto: University of Toronto Press, 2018), 36-67.

4. Zane Miller, “The Role and Concept of Neighborhood in American cities,” in *Community Organization for Social Change. A Historical Perspective*, ed. Robert Fisher and Peter Romanofsky (Westport, CT: Greenwood, 1981), 3-32.

5. David Reeder, “Neighbourhood,” in *L’Aventure les Mots de la Ville*, ed. Christian Topalov et al. (Paris: Laffont, 2010), 813.

6. Benjamin Looker, *A Nation of Neighborhoods. Imaging Cities, Communities, and Democracy in Postwar America* (Chicago: University of Chiacgo Press, 2015), 4; Miller, “The Role and Concept,” 4. The Seattle study is discussed at length in Barrett Lee et al., “Testing the Decline of Community Thesis. Neighborhood Organizations in Seattle, 1929 and 1979,” *American Journal of Sociology* 89, no. 5 (1984): 1161-1188. I am grateful to Barrett Lee for commenting specifically on the usage of “neighborhood.” Lee, personal communication, November 8, 2022.

7. For an effective use of this tool to track income inequality, see Robert Putnam and Shaylyn R. Garrett, *The Upswing. How America Came Together a Century Ago and How We Can Do It Again* (New York: Simon & Schuster, 2020).

8. J. S. Woodsworth, *My Neighbor* (Toronto: University of Toronto Press, 1972), Original published 1911.

9. Ontario. *Report of the Lieutenant-Governor’s Committee on Housing Conditions in Toronto* (Toronto: The Committee, 1934), 5; Donald Kerr, “Vancouver. A Study in Urban Geography” (Unpublished MA thesis, University of Toronto), 112; Barry W. Mayhew, Local Areas of Vancouver (Research Department, United Community Services of the Greater Vancouver Area), 2-3.

10. Alan Morley, *Vancouver. From Milltown to Metropolis*, 3rd ed. (Vancouver: Mitchell Press, 1974); Robert A. J. McDonald, *Making Vancouver. Class, Status, and Social Boundaries, 1863-1913* (Vancouver: University of British Columbia Press, 1996).

11. Herbert W. Ames, *The City Below the Hill* (Toronto: University of Toronto Press, 1972), Original published 1897; Terry Copp, *The Anatomy of Poverty. The Condition of the Working Class in Montreal, 1897-1929* (Toronto: McClelland and Stewart, 1974), 15, 17, 18, 19, 21, 23, 25; Montreal Board of Trade, “Civic Improvement League,” A Report on Housing and Slum Clearance for Montreal (Montreal: Montreal Board of Trade, 1935). On the social geography of late nineteenth-century Montreal, see Sherry Olson and Patricia Thornton, *Peopling the North American City. Montreal 1840-1900* (Montreal and Kingston: McGill-Queen’s University Press, 2011).

12. Richard Harris “The Rise of the Neighborhood in Canada, 1880s-2020s” (Toronto: University of Toronto press, forthcoming).

13. The most important limitation is that ProQuest’s *Star* database does not support keyword searches of the titles of articles, as opposed to the full text. As explained in the text, this is necessary to avoid the inclusion of many inappropriate references, muddying results.

14. The paper’s daily circulation (300,000) is slightly lower than that of the *New York Times* (340,000), although Canada’s population is only one ninth that of the United States. Both papers claim much larger “readerships.” For a recent illustration of the effective use of this database, see Giuseppe Tolfo and Brian Doucet, “Gentrification in the Media. The Eviction of Critical Class Perspective,” *Urban Geography* 42, no. 10 (2021), 1418-1439.

15. Jean W. Bradley, *A Home Across the Water. A Memoir* (Toronto: Braeward, 1996), 35.

16. “Residential zone” was occasionally used in the 1940s and 1950s but was always unusual. “Local area” was uncommon until the 1980s, when its reference was technological, to “local area networks.”

17. References to “suburb” (and its variants) remained at a fairly consistent level, but those for “slum” peaked in the 1930s and then the 1960s before falling off precipitously. By the 1980s, the pejorative connotations of the term had rendered it unacceptable in the developed world.

18. This method is reliable: a search of “the” yielded very similar results.

19. Robert J. Sampson, *Great American City. Chicago and the Enduring Neighborhood Effect* (Chicago: University of Chicago Press, 2012), 3.

20. Topalov, “Naming Process,” 60-61.

21. Raphael Fischler, “Development Controls in Toronto in the Nineteenth Century,” *Urban History Review* 36 (2007): 16-31; Peter W. Moore, “Zoning and Planning. The Toronto Experience, 1904-1970,” in *The Usable Urban Past*, ed. Alan F. J. Artibise and Gilbert Stelter (Toronto: Macmillan, 1979), 316-341.

22. “A Residential District,” *The Globe*, June 8, 1905; “Blighted Area. Shabbiness Spreading Northward,” *Globe and Mail*, August 11, 1951.

23. Moore, “Zoning and Planning”; Richard Dennis, “Toronto’s First Apartment-House Boom. An Historical Geography, 1900-1920 (Research Paper No. 177, Center for Urban and Community Studies, University of Toronto, Toronto).

24. “Distinctive Houses Amid Natural Beauty Lure Homemakers,” *The Globe*, December 18, 1925; “North Toronto a Residential Area,” *The Globe*, February 21, 1911.

25. “Ask to Operate in Home Zones,” *Globe and Mail*, June 3, 1954; “Ethnic Food. The Next Generation,” *Globe and Mail*, January 14, 2017.

26. “Business Lively in the Neighborhood of the Limestone City,” *The Globe*, October 6, 1881; “New Methodist Church for Don Neighborhood,” *The Globe*, December 11, 1922.

27. Richard Allen, *The Social Passion. Religion and Social Reform in Canada 1914-1928* (Toronto: University of Toronto Press, 1973).

28. “Making Canadians Out of Little Foreigners,” *The Globe*, October 10, 1916; “Volunteer Workers Take Leading Part in Assisting Needy,” *The Globe*, April 15, 1925.

29. The slight resurgence of “worker” from the 1990s reflects reports on the activities of “community workers.” See, for example, Raveena Aulakh, “Spate of Killings Doesn’t Faze Violence-Weary Jane-Finch,” *Globe and Mail*, December 29, 2006.

30. Judith Knelman, “Riverdale. A Neighbourhood of Contrasts,” *Globe and Mail*, December 10, 1983.

31. Douglas Webster, “Neighbourhood Planning,” *Globe and Mail*, February 16, 1970.

32. Alex Bozikovic, “The Storeys Margaret Atwood Condemns,” *Globe and Mail*, August 30, 2017.

33. Marc Choko and Richard Harris, “The Local Culture of Property. A Comparative History of Housing Tenure in Montreal and Toronto,” *Annals of the Association of American Geographers* 80, no. 1 (1990): 73-95.

34. The Author, “The Rise of the Neighborhood.”

35. Dany Fougères, “The Years of Dispersion,” in *Montreal. A History of a North American City* (Montreal and Kingston: McGill-Queen’s University Press, 2018), 300.

36. Jean-Claude Marsan, *Montréal in Evolution. Historical Analysis of the Development of Montréal’s Architecture and Urban Environment* (Montreal and Kingston: McGill-Queen’s University Press, 1981), 69; Robert Sweeney, “Aperçu d-un Effort Collectif Québécois. La Création, au Début du XXe. Siècle, d’un Marché Privé et Institutionalisé de Capitaux,” *Revie d’Histoire de l’Amérique Française* 49, no. 1 (1995): 67; Lucia Ferretti, *Entre Voisins. La Société Paroissiale en Milieu Urbain. Saint-Pierre-Apôtre de Montreal, 1848-1930* (Montréal: Boréal, 1992), 190, 181.

37. Jean-Claude Falardeau, “The Parish as an Institutional Type,” *Canadian Journal of Economics and Political Science* XV (1949): 353-367; Nancy Christie and Michael Gauvreau, *Christian Churches and Their Peoples, 1940-1965. A Social History of Religion* (Toronto: University of Toronto Press, 2010), 182.

38. Lucia Ferretti, Personal communication, June 9, 2021. I would like to take this opportunity to thank Alexandre Maltais for his assistance in surveying and interpreting the French-language planning documents for Montreal since 1944.

39. For extended discussion, see The Author, *Rise of the Neighborhood*.

40. Ray Godfrey, “The old Elm Tree Tea House,” in *Recollections of a ’Hood. Huron-Sussex from UTS to Stop Spadina*, ed. Nancy Williams and Marie Scott-Baron (Toronto: Words Indeed, 2013), 68.

41. Attempts to track block-scale activity, using “neighborly,” “neighboring,” “immediate neighborhood,” and variants on “block-face,” proved fruitless. Very few meaningful examples were found and no pattern or trend was apparent.

42. Christine Lamarre, “Quartier,” in *L’Aventure*, ed. Topalov et al., 1014; Dale Gilbert, *Vivre en Quartier Populaire. Saint-Sauveur, 1930-1980* (Québec: Septentrion, 2015); Gabrielle Roy, *Bonheur d’Occasion*. Montreal: Société d’éditions Pascal, 1945, trans. As *The Tin Flute* (Toronto: McClelland and Stewart, 1947). Roy makes 20 references to *paroisse* and 4 to *voisinage*. Writing about Québec City at the same time, Roger Lemelin also makes liberal use of *quartier* and in much the same way. Roger Lemelin, *Au Pied de la Pente Douce*. Montreal: Cercle du livre de France, 1942, trans. as *The Town Below* (New York: Reynal and Hitchcock, 1948).

43. cf. Lamarre, “Quartier.”

44. “Pour beaucoup de Montréalais, le terme ‘quartier’ se rapport à la notion traditionelle de paroisse ou de voisinage, qu’on appelle aussie milieu de ville en reference à l’espace senti et vécu par la majorité des occupants d’une communauté.”

45. Ville de Montreal. Bureau du Plan d’Urbanisme, *Plan d’Urbanisme de le Ville de Montréal* (Montreal: Ville de Montréal, 1992), 20; Ville de Montréal, *Plan d’Urbanisme de Montréal* (Montreal: Ville de Montréal, 2004), 11; Montreal. Direction des Transports, *Quartiers Verts. Guide d’Aménagement Durables des Ruse de Montréal*, trans. Maltais (Montreal: Direction des Transports, 2013), 1.4; Annick Germain, “The Montréal School. Urban Social Mix in a Reflexive City,” *Anthropologica* 55 (2013): 3.

46. Lamarre, “Quartier,” 1015.

47. Mary J. Clarke, Report of the Standing Committee on Neighborhood Work. In *Saving the Canadian City. The First Phase 1880-1920*, ed. Paul Rutherford (Toronto: University of Toronto Press, 1974), 183. Original published 1917.

48. Dorene Jacobs, “The Annex Ratepayers’ Association. Citizen Efforts to Exercise Social Choice in Their Urban Environment,” in *Citizen Participation, Canada. A Book of Readings*, ed. James A. Draper (Toronto: New Press, 1971), 288-306; The Author, *Rise of the Neighborhood*.

49. Russell Elman, *Durand. A Neighborhood Reclaimed* (Hamilton: NA Group, 2001).

50. Shlomo Hasson and David Ley, *Neighborhood Organizations and the Welfare State* (Toronto: University of Toronto Press, 1994); Looker, *Nation of Neighborhoods*, 231-258; Suleiman Osman, “The Decade of the Neighborhood,” in *Rightward Bound. Making America Conservative in the 1970s*, ed. Julian Zelizer and Bruce Schulman (Cambridge, MA: Harvard University Press, 2008); Richard White, *Planning Toronto, 1940-1980. The Planners, the Plans and their Legacies* (Vancouver: University of British Columbia Press, 2016), 320; Stephen McLaughlin, “The Challenge Ahead. Future Directions for Neighbourhood Planning,” in *Toronto Neighborhoods. The Next Ten Years*, Toronto (Toronto: City of Toronto, 1984), 4; Pierre Filion, “Rupture or Continuity. Modern and Postmodern Planning in Toronto,” *International Journal of Urban and Regional Research* 23, no. 3 (1999), 421-444.

51. Andrew Sancton, “Montreal,” in *City Politics in Canada*, ed. Warren Magnusson and Andrew Sancton (Toronto: University of Toronto Press, 1983), 73-75; Jacques Léveillée, “Pouvoir Local et Politiques Publiques à Montréal. Renouveau dans les Modalités d’Exercice du Pouvoir Urbain,” *Cahiers de recherché sociologique* 11, no. 2 (1983), 37-64.

52. Ville de Montréal. Service du Développement Culturel, *Montreal, Cultural Metropolis. Cultural Development Policy 2005-2015* (Montréal: Direction du Développement Culturel et des Bibliothèques, 2005), 23; Ville de Montréal, *Service des Infrastructures, Transportation Plan* (Montréal: Division du Développement des Transports, 2008), 42, trans. Maltais.

53. Quoted in Andy Wood, *Faith, Hope and Charity, 1500-1640* (Cambridge: Cambridge University Press, 2020), 1.

